# The African swine fever virus MGF505-1R protein recruits the cullin-RING-ligase machinery to promote p300 degradation

**DOI:** 10.1038/s44298-026-00191-8

**Published:** 2026-05-02

**Authors:** Samuel Connell, Anusyah Rathakrishnan, Joanna Wells, Linda K. Dixon, Ana Luisa Reis

**Affiliations:** 1 The Pirbright Institute, Pirbright, Woking, United Kingdom; 2 Nuffield Department of Medicine, University of Oxford, Oxford, United Kingdom

**Keywords:** Immunology, Microbiology, Molecular biology

## Abstract

African swine fever virus (ASFV) is a large, complex DNA virus that causes a haemorrhagic disease with lethality rates approaching 100%. Mechanisms of virulence are still poorly understood, but loss of members of the multigene families (MGF) is commonly observed in naturally occurring attenuated virus strains, indicating that these proteins play an important role in disease pathogenesis. Here, a suppressor of cytokine signalling (SOCS)-box like motif was identified in proteins of the MGF505 cluster. SOCS-box motifs typically recruit the Cullin-RING-ligase (CRL) machinery, a superfamily of E3 ubiquitin ligases that target proteins for proteasomal degradation. Data presented show that MGF505-1R inhibits the host innate immune responses controlled by the activation of the transcription factors IRF3 and NFκB. MGF505‑1R expression was shown to correlate with a reduction in the protein levels of the transcriptional co‑activator p300. Targeted mutations of the SOCS box motif were shown to reverse the observed IRF3 and NFκB inhibition. The data support a role for MGF505-1R in hijacking the host ubiquitin machinery to evade the host immune responses.

## Introduction

African swine fever (ASF) is a frequently lethal haemorrhagic disease of swine that poses a severe threat to food and economic security in regions dependant on pork production. Currently, licensed modified live vaccines are only available in a few countries; thus, prevention and culling are the only viable methods of control.

ASF is caused by African swine fever virus (ASFV), a large DNA virus of the *Asfarviridae* family which encapsulates a genome of up to ~190 kb encoding for over 190 open reading frames, many with unknown functions. The multigene family (MGF) genes are clusters of multiple paralogous genes found near the terminal ends of the ASFV genome. These genes can be divided into 5 clusters based upon sequence identity and amino acid length, known as MGF100, MGF110, MGF300, MGF360 and MGF505. Many genes of the MGFs, particularly those belonging to MGF360 and MGF505, are known to play roles in virulence and host immune response modulation^1–10^.

The MGF505 gene cluster, named so as individual genes code for ~505 amino acids, comprises up to 10 members, depending on the virus isolate^11^. They share conserved structural features including predicted ankyrin repeats^12^. Ankyrin repeats are present in a wide range of functionally diverse proteins, mediating protein–protein interactions. Whilst their structure is well conserved, the surface residues are highly variable, allowing specific interactions with a broad range of targets^13^. It was proposed that the MGF505 (and MGF360) functional diversity is associated with motifs within the ankyrin repeats under variable evolutionary selection, representing different interactions with host proteins^12^.

Of the MGF505 proteins, MGF505-7R has been the most studied and was shown to inhibit both the induction of type I interferon (IFN), cellular responses to IFN^14^ and inflammasome activation^15^. The mechanisms underlying these functions were further explored by identifying interacting host protein targets. They include the inhibitor of κB kinase (IKK)α^16^ and p65^17^, a central kinase and the main transcription factor of the NFκB pathway, respectively. Furthermore, several key molecules involved in the induction of IFN such as the stimulator of interferon genes (STING)^18^, TANK-binding kinase 1 (TBK1) and IFN regulatory factor (IRF)7^19^ were shown to be targeted for degradation in cells expressing MGF505-7R. Translocation of IRF3 into the nucleus was also impaired in these cells^16^. The inhibition of the cellular responses to IFN by MGF505-7R appears to rely on its interaction with Janus kinase (Jak)1 and Jak2, leading to their degradation^20^, and with IRF9, to inhibit the nuclear translocation of the interferon-stimulated gene factor 3 (ISGF3)^21^. Interestingly, MGF505-11L^22^, MGF505-6R^23^ and MGF505-2R^24^ were also shown to interact with STING; the first two of these resulted in STING degradation. MGF505-3R and MGF505-4R, were shown to inhibit IFN induction by inducing degradation of TBK1^25^ or possibly by interacting with TRAF3^26^, respectively.

Here, we identified a suppressor of cytokine signalling (SOCS)-Box like motif in all proteins of the MGF505 cluster. These motifs are present in many host proteins and are known to recruit the cullin-RING-ligase (CRL) machinery to facilitate the targeting of proteins for ubiquitination and degradation. Additionally, some viral proteins exploit the host CRL machinery to induce the degradation of specific targets they bind to^27^. The CRL is a modular E3 ubiquitin ligase complex formed of a substrate-binding protein, a scaffold cullin (Cul) protein, a variable adaptor protein, a RING E3 ligase, and an E2 ligase. The specific classes of each protein within the complex recruited by the substrate-binding protein are dictated by the amino acid motifs present. The SOCS-Box motif recruits the ElonginB (EloB) ElonginC (EloC) heterodimer adaptor, Cul5 scaffold, RBX2 RING and E2 ligase^28,29^. We show that MGF505-1R recruits both EloB and Cul5, and that mutations of the SOCS-Box motif reduce these interactions. We also show that MGF505-1R, -2R and -3R inhibit the IRF3 and NFκB dependent signalling pathways, but do not inhibit their translocation into the nucleus or transcriptional activity. Further investigation revealed that MGF505-1R targets the p300 transcription co-activator for degradation, which may represent a key mechanism through which MGF505‑1R inhibits IRF3 and NF‑κB signalling.

## Results

### MGF505-1R, -2R and -3R proteins inhibit the IRF3 signalling pathway

**Fig. 1:**
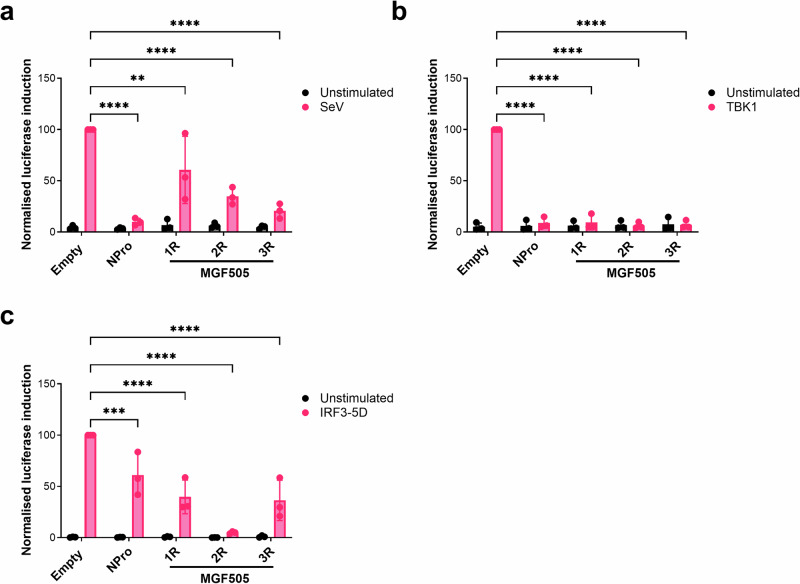
MGF505-1R, -2R and -3R proteins inhibit IRF3 mediated signalling. HEK293T cells were transfected with a plasmid containing a luciferase reporter gene under the control of IRF3 regulatory elements of the IFNβ promoter. Cells were co-transfected with plasmids expressing the indicated MGF genes fused to a V5-tag, or empty vector or NPro as controls. Co-transfection with plasmids expressing renilla luciferase (**a** and **b**) or βgal (**c**) were used as internal control. Cells were stimulated with: Sendai virus (200 HAU/mL for 24 h) (**a**); or transfected to exogenously express TBK1.flag (**b**) or constitutively active IRF3-5D (**c**). Luciferase data were normalised to the empty-vector control and the graphs show results from three independent experiments. For statistical analysis two-way ANOVA Dunnett’s multiple comparison test was performed, comparing stimulated empty vector to stimulated MGFs. ****p ≤ 0.0001, ***p ≤ 0.001, **p ≤ 0.01.

We used a firefly luciferase reporter construct under the control of the IRF3 response element of the IFNβ promoter, to test the ability of MGF505-1R, -2R, -3R proteins to control type I IFN responses. These were chosen as we have already characterised a panel of ASFV deletion mutants comprising these genes^10^. HEK293T cells were transfected with constructs expressing the reporter gene, genes of interest, and, in the case of exogenously stimulated cells, plasmids coding for stimulants, TBK1 or a constitutively active IRF3 (IRF3-5D). Unstimulated cells were transfected with equal quantity of empty vector pcDNA3.1 to ensure the total amount of DNA transfected was consistent with stimulated cells. Sendai virus (SeV) was used to stimulate the IRF3 pathway^30–32^. A construct expressing the classical swine fever virus NPro protein, a known IRF3 inhibitor, was used as a positive control for inhibition^33,34^. Using this assay, we observed a significant decrease in relative firefly luciferase expression in the presence of MGF505-1R, -2R and -3R and positive control when cells were stimulated with SeV (Fig. 1a), TBK1 (Fig. 1b) and IRF3-5D (Fig. 1c), indicating that these MGF505 proteins inhibit IRF3 signalling at the transcription factor level or below.

**Fig. 2:**
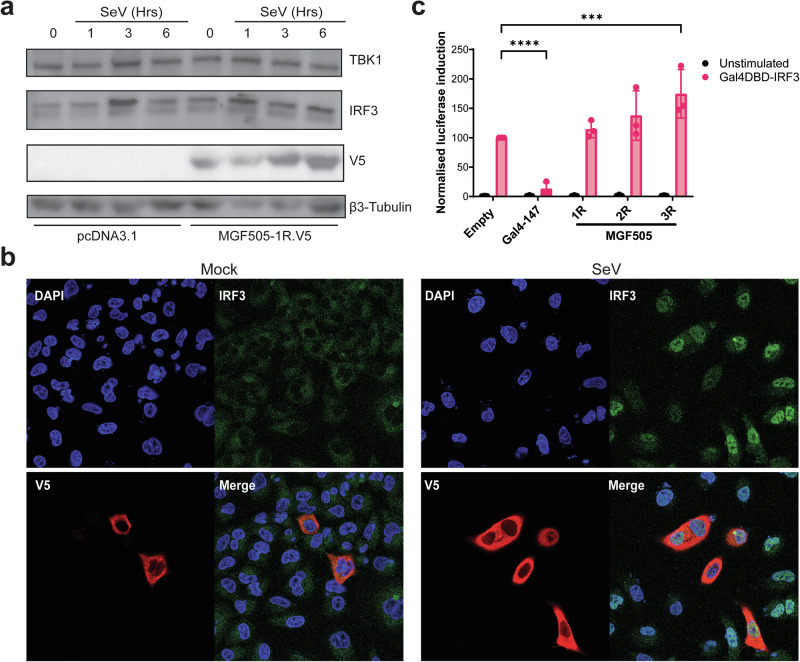
MGF505-1R inhibits IRF3 after its translocation into the nucleus. **a** Western Blotting of HEK293T cell lysates expressing pcDNA3.1 empty vector or MGF505-1R.V5. Cells were stimulated with Sendai virus for different time periods (hours) indicated in the top bar. Protein levels in cell extracts were investigated by probing blots of SDS/PAGE gels with relevant antibodies as indicated on the right side. Tubulin was used as endogenous loading control. **b** Immunofluorescent staining of transfected A549 cells showing predominant IRF3 cytoplasmic expression in non-stimulated cells (left panel) and successful translocation of IRF3 to the nucleus upon stimulation with Sendai virus (200 HAU/ml) for 4 h (right panel). Cells were transfected with a plasmid expressing MGF505-1R.V5 and the V5-tag was stained red with mouse anti-V5 followed by goat anti-mouse 568 secondary antibodies. IRF3 was stained green with rabbit anti-IRF3, and anti-rabbit 488 secondary antibodies. DAPI was used to stain the nucleus blue. **c** HEK293T cells transfected with constructs expressing Gal4-luciferase reporter gene and βGal internal control. Cells were co-transfected with plasmids expressing V5-tagged MGF505-1R, -2R or -3R, and stimulated cells transfected with Gal4DBD-IRF3, or in unstimulated cells co-transfected with equal amounts of pcDNA3.1 empty vector. Luciferase data were normalised to the empty-vector control and the graph shows results from three independent experiments. For statistical analysis, two-way ANOVA Dunnett’s multiple comparison test was performed comparing stimulated empty vector to stimulated MGFs. ****p ≤ 0.0001, ***p ≤ 0.001.

We next investigated the impact of MGF505-1R upon the integrity of the pathway via western blotting. HEK293T cells were transfected with constructs including the empty vector control or expressing MGF505-1R protein tagged with V5 at the C-terminus (MGF505-1R.V5) and stimulated with SeV for different time periods. Although some differences were observed across different time points, MGF505-1R.V5 had no obvious impact on the endogenous levels of IRF3 or TBK1, relative to control cells transfected with the empty vector (Fig. 2a). Confocal microscopy was used to probe for a potential impact of MGF505-1R.V5 on nuclear translocation of IRF3. A594 cells were transfected with constructs including the empty vector control or expressing MGF505-1R.V5, and infected with SeV for 4 h or mock infected. At least 50 cells were counted per condition. As expected in non-stimulated cells (mock stimulated), IRF3 is localised in the cytoplasm. Following SeV stimulation, nuclear translocation of endogenous IRF3 was observed in both the control cells and the MGF505-1R.V5 expressing cells (Fig. 2b).

Next, we tested the ability of MGF505-1R, -2R and -3R to inhibit IRF3 transcriptional activity, by using an alternative luciferase assay in which the expression of firefly luciferase is under the control of the GAL4 promoter. Exogenous expression of Gal4-IRF3-65-427, a fusion protein of the Gal4 DNA binding domain (DBD) and the transcription activation domain (TAD) of IRF3, was used to stimulate this alternative GAL4 controlled luciferase. In this assay, induction of firefly luciferase is dependent on the IRF3 transcriptional activity but independent of its ability to bind to DNA. As expected, the Gal4 DBD domain alone (Gal4-147) competes with the Gal4-IRF3-65-427 fusion protein to bind to the promoter, thus reducing luciferase activity. Interestingly, we observed no reduction of luciferase activity in cells transfected with plasmids expressing any of the three MGF505 proteins (Fig. 2c). This result indicates that these proteins do not directly inhibit IRF3 transcriptional activity.

### MGF505-1R, -2R and -3R proteins inhibit the NFκB signalling pathway

**Fig. 3:**
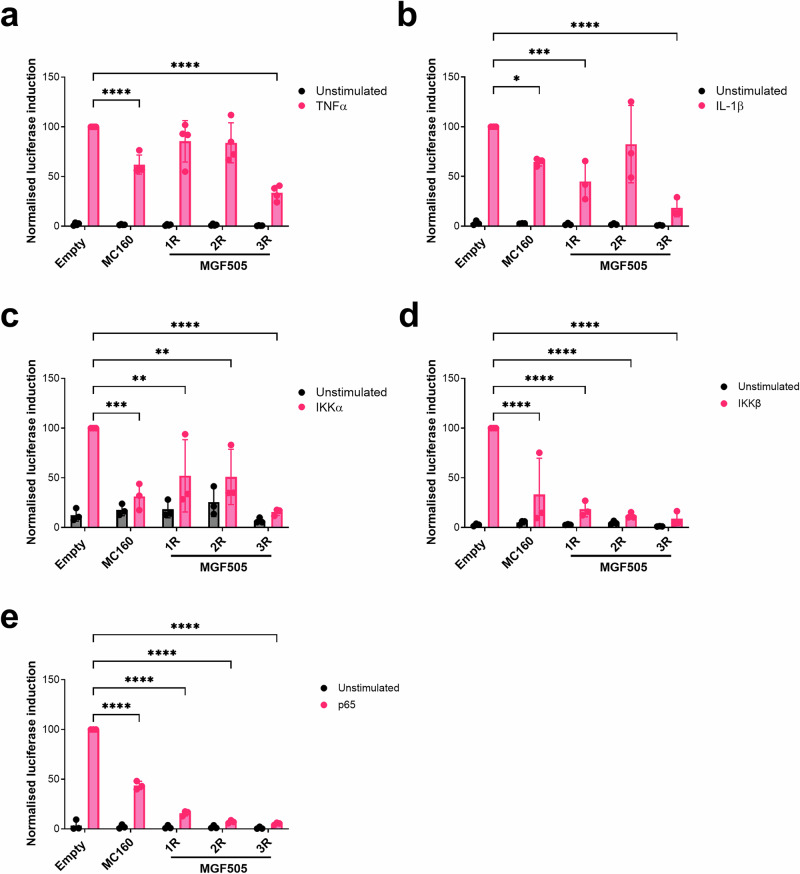
MGF505-1R, -2R and -3R proteins inhibit NFκB-mediated signalling. HEK293T cells were transfected with a plasmid containing a luciferase reporter gene under the control of NFκB regulatory elements of the IFNβ promoter. Cells were co-transfected with plasmids expressing the MGF genes indicated fused to a V5 tag, or empty vector or MC160 as controls. Cells were co-transfected with plasmids expressing renilla luciferase as an internal control. Cells were stimulated with: TNFα (**a**) and IL-1β (**b**) or transfected to exogenously express IKKα.flag (**c**), IKKβ.flag (**d**) or p65-GFP (**e**). Luciferase data were normalised to the empty-vector control and the graphs show results from three or four (TNFα) independent experiments. For statistical analysis two-way ANOVA Dunnett’s multiple comparison test was performed comparing stimulated empty vector to stimulated MGFs. ****p ≤ 0.0001, ***p ≤ 0.001, **p ≤ 0.01, *p ≤ 0.05.

To investigate the effect of the MGF505-1R, -2R and -3R proteins on activation of the NFκB pathway, we stimulated cells using TNFα, or IL-1β to activate the TNFα receptor or the IL-1β receptor, respectively. MC160, a known inhibitor of the NFκB pathway was used as a positive control^35,36^. Cells were co-transfected with a luciferase reporter construct containing an upstream NFκB binding motif. This revealed that some of the MGF505 proteins investigated strongly inhibited the pathway, particularly following stimulation with IL-1β (Fig. 3a, b). To determine the specific target inhibited by the MGF505 proteins, key components of the pathway were exogenously co-expressed with each of the MGF family proteins. Plasmids expressing IKKα and IKKβ, the key kinases which phosphorylate IκBα, leading to its degradation and release of the NFκB transcription factor^37,38^, induced luciferase activity driven by the reporter construct. Each of the MGF505 genes tested significantly inhibited this induction (Fig. 3c, d). Finally, expression of all three MGF505 proteins showed they also significantly inhibited activation induced by transient expression of p65, the main transcription factor of the canonical NFκB pathway. This suggests that the MGF505 proteins tested inhibited NFκB activation at the p65 level, or below (Fig. 3e).

**Fig. 4:**
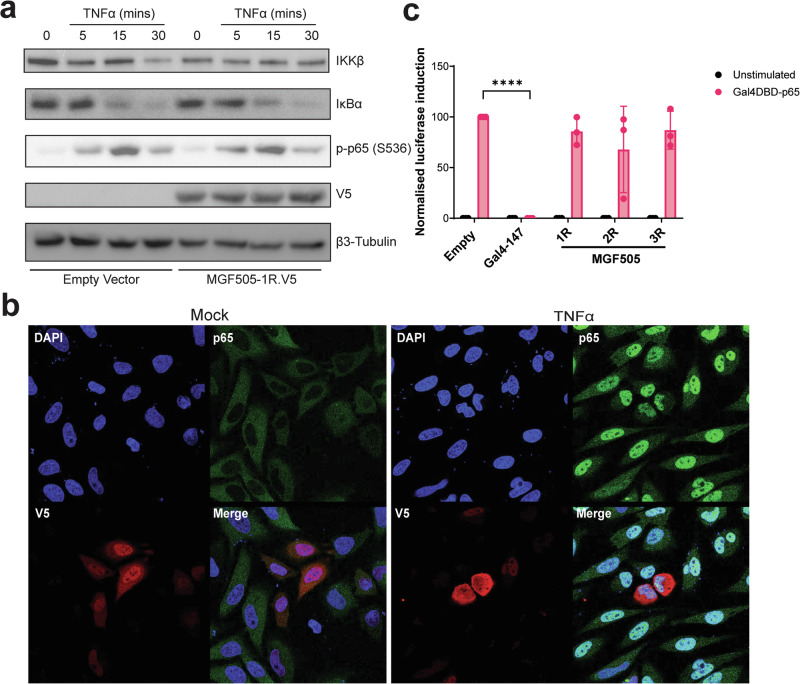
MGF505-1R inhibits the NFκB pathway after p65 translocation into the nucleus. **a** HEK293T cells were transfected with pcDNA3.1 empty vector or MGF505-1R.V5. Cells were stimulated with TNFα for the time period indicated in the top bar. Endogenous protein levels (see right-hand side) were assessed by western blotting following SDS-PAGE separation. Westerns blots are representative of experiments performed in duplicate. **b** Confocal immunofluorescence showing Hela cells transfected with a plasmid expressing MGF505-1R fused to V5-tag, stimulated with TNFα for 20 minutes, and probed for V5 (red), and endogenous p65 (green). Blue shows DAPI nuclear staining. **c** HEK293T cells were transfected with constructs expressing Gal4-luciferase reporter gene and renilla internal control. Cells were co-transfected with MGFs, or Gal4-147 as control. Stimulated cells were co-transfected with Gal4DBD-p65, or in unstimulated cells co-transfected with equal amounts of pcDNA3.1 empty vector. Luciferase data were normalised to the empty-vector control and the graph shows results from three independent experiments. For statistical analysis, two-way ANOVA Dunnett’s multiple comparison test was performed comparing stimulated Empty Vector to stimulated MGFs. ****p ≤ 0.0001.

We next used TNFα to activate the NFκB pathway and tested IκBα degradation and p65 phosphorylation by western blotting (Fig. 4a). Similar results were obtained in cells transfected with the empty vector or expressing MGF505-1R.V5. As expected, increased levels of phosphorylated p65 were observed at 5 min following stimulation, reaching a peak at 15 min, and decreasing by 30 min. Levels of IκBα were reduced at 15 and 30 min compared to 0 and 5 min. Exogenously expressed, MGF505-1R.V5 did not inhibit these crucial steps of the pathway activation. Confocal microscopy of HeLa cells transfected with the construct expressing MGF505-1R.V5 compared to the empty vector, revealed that the expression of the viral protein did not inhibit the nuclear translocation of p65 post-stimulation with TNFα for 30 min (Fig. 4b). Interestingly, in HeLa cells, a higher proportion of cells showed MGF505-1R localised to both the cytoplasm and the nucleus (Fig. 4b) as compared with A549 cells in which MGF505-1R was predominantly cytoplasmatic (Fig. 2b). These differences in localisation may reflect different interactions with host proteins between these cell lines.

The results above suggest that the inhibition caused by MGF505-1R expression may occur by a direct impact on the NFκB transcriptional activity, by interfering with the transcription cofactor machinery or by blocking the transcription factor binding to the promoter. To investigate these possibilities, HEK293T cells were transfected with a construct expressing firefly luciferase under the control of GAL4 promoter, in a similar manner described for IRF3. Cells were stimulated by the exogenous expression of Gal4 DBD fused to p65 known as Gal4DBD-p65. In this system, the Gal4 DBD domain brings the fusion protein into proximity to the promoter, whilst p65 provides the required TAD to activate transcription of the luciferase reporter gene. We observed no inhibition of luciferase stimulation by Gal4DBD-p65 in cells expressing MGF505-1R, -2R or -3R (Fig. 4c). This indicates that the inhibition of p65 transactivation activity is not the mechanism of NFκB inhibition. In control cells as expected, the overexpression of Gal4-147 resulted in inhibition of the luciferase stimulation by Gal4DBD-p65, most likely by competing with the fusion protein for binding to the GAL4 promoter. Taken together these results indicate that the inhibition of both IRF3 and NFκB-dependent pathways by MGF505-1R occur at the level of the promoters, either by inhibiting transcription factor binding to DNA or the recruitment of other proteins necessary to the assembly of the transcription complex.

### MGF505s contain a putative SOCS/VHL box-like motif

Since previous studies have suggested that MGF505-7R^19,20^ and MGF505-11L^22^, are associated with proteasome-dependent host protein degradation, we explored the presence of specific amino acid motifs able to recruit the CRL machinery in members of the MGF505 proteins. Recruitment of the CRL machinery by MGF505 proteins could promote ubiquitination and degradation of specific substrate proteins also bound by individual MGF505 proteins. Since MGF proteins are divergent in sequence they may potentially bind different substrate proteins to target for degradation.

**Fig. 5:**
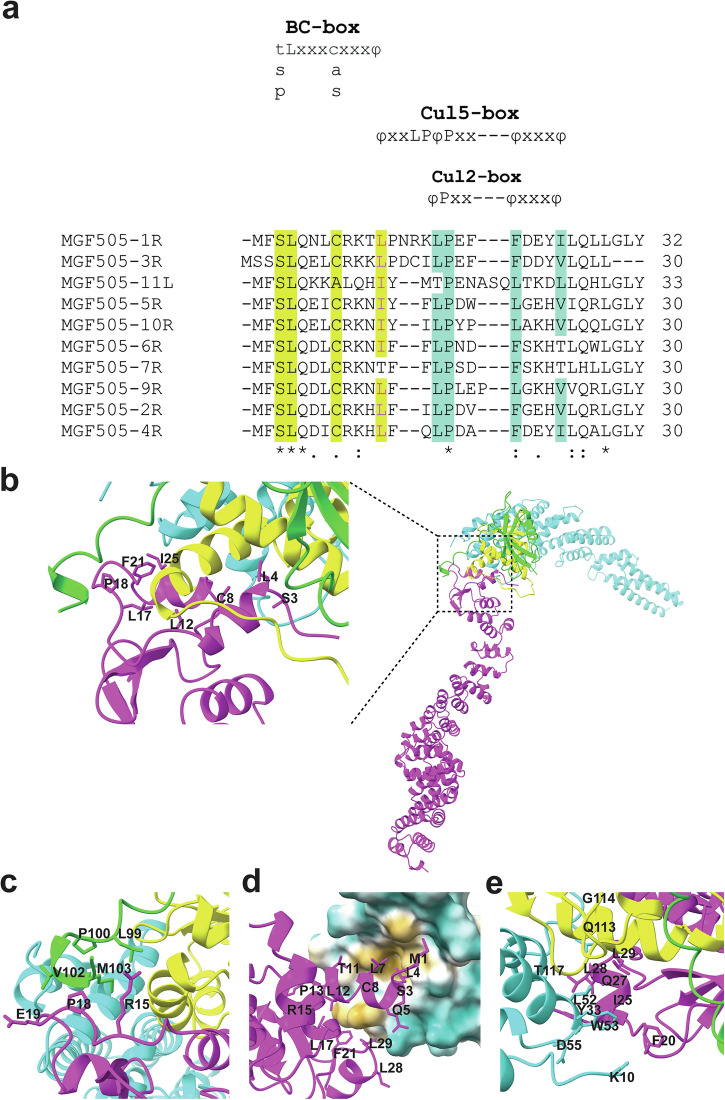
MGF505-1R contains a VHL/SOCS box motif. **a** Alignment of MGF505 proteins, residue homology is shown beneath. * indicates similarity, **:** indicates highly conserved, indicates conserved. The BC-box and Cul5/Cul2 boxes consensus sequences are shown at top of the alignments. Conserved residues in the BC-box are highlighted in yellow and conserved residues in the Cul5/Cul2 boxes are highlighted in cyan. Overlapping residues are highlighted in yellow with red text. **b** AlphaFold3 model of the complex MGF505-1R (magenta)/ EloC (yellow)/ EloB (green)/ Cul5 (cyan), zoomed in on the VHL/SOCS box and showing the residues highlighted in the alignment in (**a**). **c** Residues predicted to mediate the interaction between MGF505-1R (magenta) and EloB (green). **d** Residues in MGF505-1R (magenta) predicted to interact with EloC (hydrophobic residues are represented in brown and hydrophilic residues are represented in blue). **e** Residues predicted to mediate the interaction between MGF505-1R (magenta) and Cul5 (cyan).

Sequence alignment against CRL recruitment motifs revealed the presence of an N-terminal VHL/SOCS-box-like motif in the MGF505 cluster. Although highly similar, the VHL-Box and SOCS-Box can be further divided into constituent components. A BC-Box, which recruits EloB and EloC, and a Cul binding region. A Cul2-Box is archetypal of a VHL-Box, whereas a Cul5-Box is typical of an SOCS-Box. The presence of the BC-box ([TSP]Lxxx[CAS]xxxφ) in the MGF505 proteins was clear from homologous residues in alignment with the VHL/SOCS-Box consensus sequence, whereas whether the Cul binding region was Cul2-Box (φPxxφxxxφ) or Cul5-Box (φxxLPφPxxφxxxφ) remained unclear, as a hydrophobic residue is shared between the BC-box and the Cul5-box (Fig. 5a).

Structural modelling of the complex between MGF505-1R, EloB, EloC and Cul5 (Fig. 5b) or Cul2 (not shown) using AlphaFold3^39^ supports the presence of a SOCS/VHL box motif, with a predicted template modelling (pTM) score of 0.62 and 0.65 and a interface predicted template modelling (ipTM) score of 0.68 and 0.7, respectively. For the MGF505-1R/EloB/EloC/Cul5 complex, the model predicts that the interaction between MGF505-1R with EloB is mediated by R15, P18 and E19 in the viral protein and L99, P100, V102 and M103 in EloB (Fig. 5c). Interestingly, V102 and M103 were previously shown to mediate binding of EloB to SOCS2^40^ indicating that MGF505-1R may occupy the same binding site as SOCS2. For the interaction of MGF505-1R with EloC, L4 and C8, which are buried in a hydrophobic pocket in EloC, are predicted to play an important role (Fig. 5d), in a similar manner to L163 and C167 in SOCS2^41^. The SOCS2/Cul5 binding is mediated by a ring-to-ring interaction between W53 in Cul5 and P184 in SOCS2^40^. In the MGF505-1R/EloB/EloC/Cul5 complex, the “ring” is provided by F20 (Fig. 5e), not P18 as it would be expected from the alignment with the Cul5 box consensus (Fig. 5a).

### MGF505-1R interacts with EloB and Cul5

**Fig. 6:**
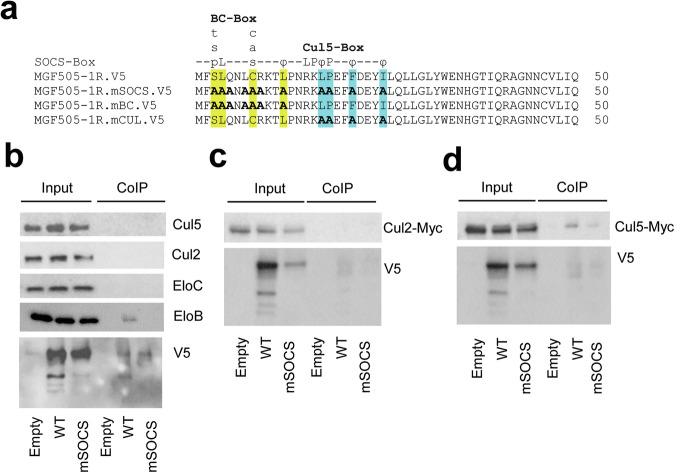
MGF505-1R interacts with EloB and Cul5. **a** Mutations of the putative VHL/SOCS-Box of MGF505-1R. Residues highlighted in yellow correspond to those that are homologous with key residues of the BC-Box, residues highlighted in cyan correspond to the Cul2/5-Box. Residues in bold are mutations. **b**–**d** CoIP to investigate interacting partners of MGF505-1R using lysates from HEK293T cells transfected with empty vector or, V5-tagged viral genes MGF505-1R.V5 (WT), MGF505-1R.mSOCS.V5. A sample of the lysate was taken as “Input”. The remaining lysate was incubated with magnetic beads covalently linked to anti-V5 antibodies to pulldown complexes containing V5-tagged proteins (CoIP). Samples were analysed by western blotting following SDS-PAGE separation. Bands corresponding to V5-tagged MGF505-1R.V5 and MGF505-1R.mSOCS.V5 were observed in the Input and CoIP. **b** Input and CoIPs were probed with antibodies against endogenous Cul5, Cul2, EloC and EloB. **c** Cells were additionally co-transfected with plasmids expressing Cul2.Myc and an anti-V5 antibody was used to pulldown complexes containing V5-tagged proteins (CoIP). Lanes containing input and CoIP were probed with anti Myc-antibodies. **d** Cells were additionally co-transfected with plasmids expressing Cul5.Myc and an anti-V5 antibody was used to pulldown complexes containing V5-tagged proteins (CoIP) Input and CoIP were probed with anti-Myc antibodies.

To assess the functionality of these SOCS/VHL motifs, co-immunoprecipitation (CoIP) was used to investigate if MGF505-1R.V5 interacted with members of the CRL machinery. Additionally, we generated mutant variants of MGF505-1R.V5 with key residues that aligned with the SOCS/VHL consensus sequence mutated to alanine (Fig. 6a). In one mutant, the full motif was disrupted by substitution of 7 residues with Alanine in the BC box and 4 residues in the Cul5 box (MGF505-1R.mSOCS.V5); furthermore, to separately determine the role of the BC-Box and Cul2/5-Box motifs, we generated mutants disrupting these regions only: MGF505-1R.mBC.V5 with 7 residues in the BC box substituted with Alanine and MGF505-1R.mCUL.V5 with 4 residues in the Cul2/5 box substituted respectively. HEK293T cells were transfected with either pcDNA3.1 empty control plasmid, wild-type MGF505-1R.V5 (WT), or variants with mutations in the key residues of the putative VHL/SOCS-Box-like motif (mSOCS). According to the model shown in Fig. 5, mutations in S3, L4, Q5, L7, L12, L17 and F21 would disrupt interaction with EloC, P18 would disrupt interaction with EloB and I25 with Cul5. Only the mutation in R9 is not predicted to have an impact on the complex formation.

For the CoIP, cells were incubated in DSP crosslinking agent prior to cell lysis, to stabilise protein–protein interactions. We immunoprecipitated the V5-tagged WT MGF505-1R.V5 protein and mutant variant MGF505-1R.mSOCS.V5 using epoxy beads covalently linked with anti-V5 antibodies. The amounts of V5‑tagged proteins recovered in the pull‑down were comparatively low, however we observed a stronger band at the expected molecular weight of the full-length MGF505-1R at 62 kDa, but also bands at lower molecular weights. These low levels of recovered bait protein made the co-precipitation assays difficult to interpret. Nevertheless, we observed co-immunoprecipitation of WT MGF505-1R.V5 with endogenous EloB, but were unable to pulldown EloC, Cul2 or Cul5. We were also unable to co-immunoprecipitate any of those proteins from cells transfected with the empty vector control, or the mutant variant MGF505-1R.mSOCS.V5 (Fig. 6b).

Following co-transfection of plasmids expressing Cul2.Myc and Cul5.Myc, with the MGF505-1R.V5 co-precipitation was observed with Cul5.Myc but not with Cul2.Myc (Fig. 6c, d). Additionally, a faint band was observed in the co-precipitation experiment between the MGF505-1R.mSOCS.V5 and Cul5.Myc (Fig. 6d).

To further investigate possible interactions between MGF505-1R.V5 and EloC, cells were transfected with a plasmid expressing EloC fused to a T7 tag, together with either empty vector control, WT MGF505-1R.V5 or mutant MGF505-1R.mSOCS.V5. We were unable to co-immunoprecipitate either WT or mutant MGF505-1R.V5 with overexpressed EloC.T7 (not shown). This was unexpected as the model predicts an interaction with EloC. However, since EloB plays an important role in stabilising the CRL complex^41^, it is possible that the interaction between MGF505-1R and EloC is influenced by the amount of EloB expressed in the cells.

Taken together, these results suggest that the SOCS/VHL-Box-like motif in MGF505-1R is functioning as a SOCS-Box, as the protein can interact with EloB and Cul5.

### Mutations of MGF505-1R’s SOCS-Box alleviate inhibition of the IRF3 & NFκB Pathways

**Fig. 7:**
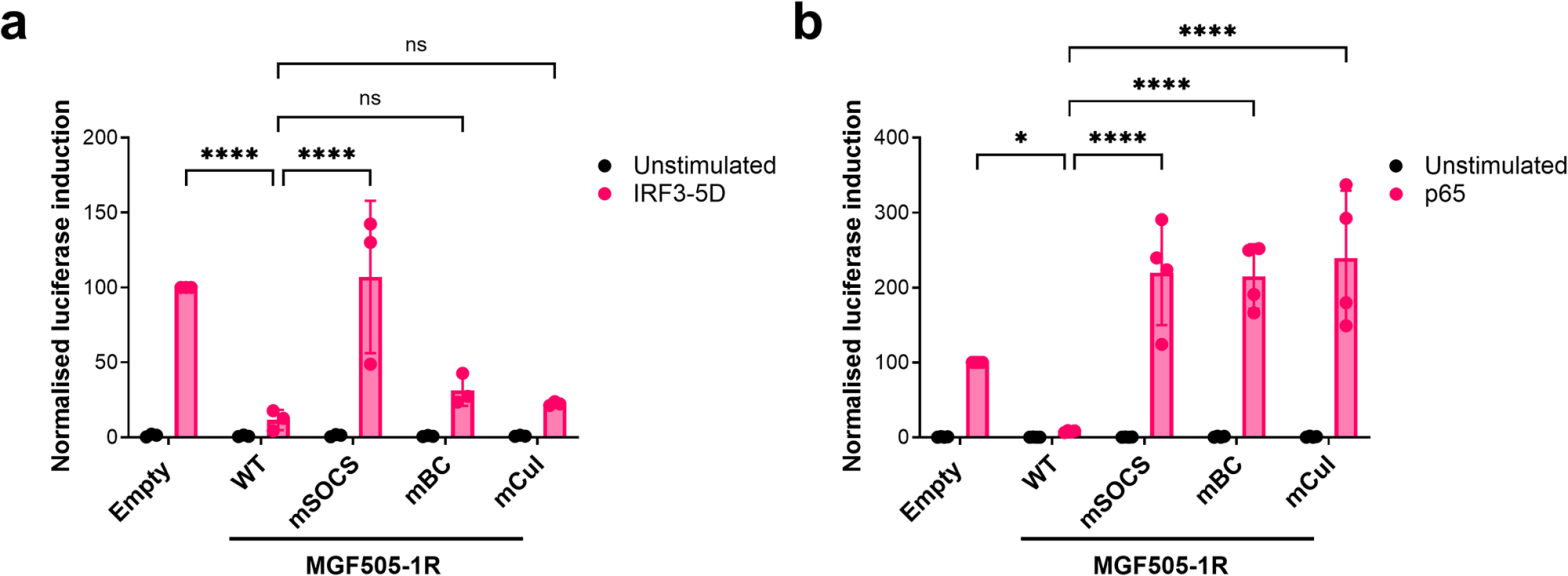
Mutations in the SOCS box of MGF505-1R alleviate inhibition of IRF3 and NFκB pathways. HEK293T cells were transfected with firefly luciferase reporter plasmids under the control of either **a** IRF3 or **b** NFκB response elements, and with control plasmids expressing βgal (**a**) or renilla luciferase (**b**). Cells were co-transfected with WT MGF505-1R.V5 or its mutant variants (MGF505-1R.mSOCS.V5, MGF505-1R.mBC.V5, MGF505-1R.mCUL.V5). Cells were co-transfected with plasmids expressing **a** IRF3-5D or **b** p65.GFP to stimulate IRF3 or NFκB-driven luciferase reporter constructs, respectively. Luciferase data were normalised to the empty-vector control and the graphs show results from three (IRF3) or four (p65) independent experiments. For statistical analysis, two-way ANOVA Tukey’s multiple comparison test was performed comparing stimulated wild-type MGFs to stimulated mutant MGFs. ****p ≤ 0.0001, *p ≤ 0.05.

Using the same luciferase reporter assays described above, we then investigated the impact of introducing mutations into the SOCS-Box of MGF505-1R.V5 on inhibition of the IRF3 and NFκB pathways (Fig. 7a, b). HEK293T cells were transfected with plasmids containing firefly luciferase reporter gene under the control of either IRF3 or NFκB regulatory elements of the IFN-β promoter, and plasmids expressing βGal (for IRF3-5D stimulation) or renilla luciferase (for p65-GFP stimulation) were used as internal controls. Cells were co-transfected with pcDNA3.1 empty vector control, or WT MGF505-1R.V5 or mutant variants MGF505-1R.mSOCS.V5, MGF505-1R.mBC.V5 or MGF505-1R.mCUL.V5. Co-transfection of plasmids expressing constitutively active IRF3-5D or p65-GFP were used to stimulate the IRF3 and NFκB pathways as above.

Assays for activation of the IRF3 pathway, showed that mutation of the full SOCS-Box motif (MGF505-1R.mSOCS.V5) reduced the inhibitory activity compared with the wild type MGF505-1R protein suggesting that the mutated sequence was critical for the inhibition (Fig. 7a). The MGF505-1R.mBC.V5 and MGF505-1R.mCUL.V5 mutants retained inhibitory function of IRF3 luciferase, suggesting that mutation of these regions alone was insufficient to alleviate the IRF3 inhibitory function of MGF505-1R (Fig. 7a).

The mutation of the entire SOCS-Box-like motif found in MGF505-1R alleviated the inhibition of the NFκB-dependant luciferase reporter when stimulated by exogenous expression of p65-GFP. In contrast to the IRF3 pathway, it was clear that the introduction of mutations disrupting the BC-Box region of the motif (MGF505-1R.mBC.V5) or the Cul5-Box region (MGF505-1R.mCUL.V5) also reversed the inhibitory impact of MGF505-1R on the NFκB pathway (Fig. 7b).

### MGF505-1R reduces endogenous levels of p300

Our results suggested that the inhibition of both NFκB and IRF3 pathways by MGF505-1R occurred post-activation of the relevant transcription factors and that nuclear translocation of the transcription factors was unimpaired. Thus, the target for inhibitory activity should be further downstream in the pathway. Binding of the transcription factors to the IFNβ promoter requires the further recruitment of proteins to the complex. These include histone acetyl transferase proteins such as CREB binding protein (CBP), or its paralog p300. These co-activators aggregate directly at the promoter, targeting H3/H4 histones for acetylation, and are considered essential for transcriptional synergy^42–46^. We therefore tested if transcriptional cofactors common to both IRF3 and NFκB pathways such as p300 and CBP may be potential targets for MGF505-1R inhibitory activity.

**Fig. 8:**
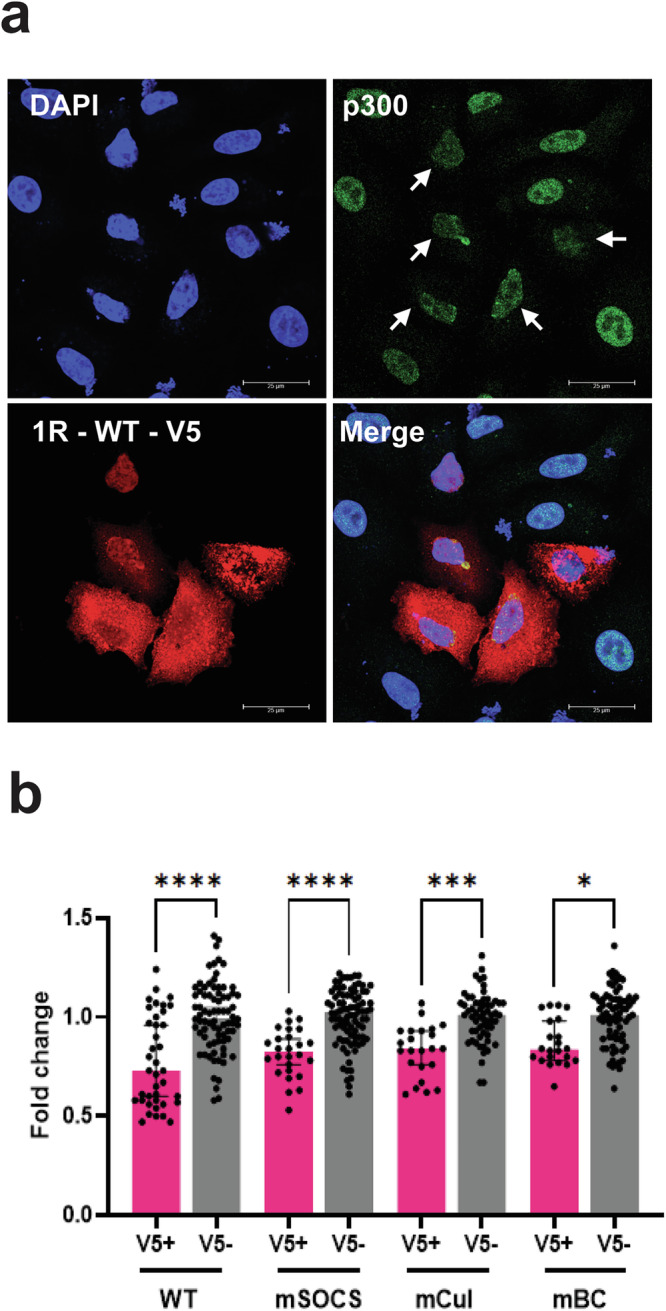
MGF505-1R reduces endogenous p300 protein levels. **a** Confocal immunofluorescence showing A549 cells transfected with a plasmid expressing WT MGF505-1R fused to V5-tag, and probed for V5 (red), and endogenous p300 (green). Blue shows DAPI nuclear staining. **b** Quantification of the p300 fluorescence signal in A549 cells transfected with WT MGF505-1R.V5 (*n* = 118) or its mutant variants MGF505-1R.mSOCS.V5 (*n* = 114), MGF505-1R.mBC.V5 (*n* = 83) and MGF505-1R.mCUL.V5 (*n* = 97). Values were normalised to the mean p300 intensity of the V5‑negative cells within each confocal image. For statistical analysis, two-way ANOVA Tukey’s multiple comparison test were performed comparing V5 positive and V5 negative cells. ****p ≤ 0.0001, ***p ≤ 0.001 *p ≤ 0.05.

A549 cells were transfected with pcDNA3.1 empty vector as control, or WT MGF505-1R.V5, or mutant variants; MGF505-1R.mSOCS, MGF505-1R.mBC.V5 or MGF505-1R.mCUL.V5. Cells were then fixed and stained for both V5 and p300. We observed that cells expressing MGF505-1R exhibited reduced p300 levels (Fig. 8a, Supplementary Fig. 1). The reduction in amount of p300 could be explained by MGF505-1R targeting p300 for ubiquitin-mediated degradation as predicted from the presence of the SOCS-Box motif. This might involve a direct interaction between MGF505-1R and p300. However, and although we occasionally observed areas of overlap between the MGF505-1R and p300 signals, we were unable to establish clear or consistent co-localisation (Fig. 8a, Supplementary Fig. 1). Additionally, following transfection of a plasmid expressing MGF505-1R.V5 into cells and immunoprecipitation using anti-V5 antibody, we were unable to show an interaction between MGF505-1R and endogenous p300 (data not shown). This suggests that the interaction between the proteins may be transient and/or mediated by additional interacting partners. To assess potential differences in p300 expression in cells transfected with WT MGF505‑1 R.V5 and its mutant variants, we quantified p300 fluorescence intensity using the analysis software Imaris. This analysis revealed a clear reduction in p300 intensity in cells expressing WT MGF505‑1 R. Interestingly, a decrease was observed for all mutant variants tested. Although there was a trend toward lower p300 levels in cells expressing the wild‑type MGF505-1R protein compared with the mutants, this difference did not reach statistical significance (Fig. 8b). Therefore, the mutant MGF505-1R proteins may still retain the ability to degrade p300 to some extent. We therefore cannot exclude the possibility that additional mechanisms (beyond the observed degradation of p300) may contribute to the inhibitory effects of MGF505‑1R on IRF3 and NFκB pathways. These may include the degradation of additional host proteins. Unbiased approaches such as mass spectrometry could help identify these potential targets.

## Discussion

Here, we investigated genes of the ASFV MGF505 cluster and revealed functional mechanisms that the MGF505-1R, -2R and -3R proteins exert on the IRF3 and NFκB signalling pathways. Our results indicated that the inhibition occurs at a point in the pathways post-activation of the transcription factors IRF3 or NFκB. By utilising promoter reporter assays, we demonstrated that exogenous expression of MGF505-1R, -2R, and -3R was capable of inhibiting both the IRF3 and NFκB signalling pathways at the transcription factor level. Importantly, inhibition of both pathways was relieved using an alternative GAL4 luciferase assay system, where the induction of luciferase is solely dependent on the transcriptional activity of the relevant transcription factors. This strongly suggests that the inhibition of IFN induction is not via blocking the transcriptional activity of the IRF3 or NFκB factors directly, nor by blocking transcription more broadly. We then focused on MGF505-1R, and showed that it did not have an obvious impact on the endogenous levels of key components within the signalling pathways and did not inhibit nuclear translocation of either IRF3 or NFκB following upstream pathway stimulation. Others have recently shown that MGF505-2R interacts with STING to inhibit IFNβ expression^24^. Here, we did not specifically probe the cGAS-STING pathway, but instead used SeV infection to induce retinoic acid-inducible gene I (RIG-I)-like receptors (RLR) signalling to activate IRF3. Nevertheless, it is expected that by impacting on IRF3, MGF505-2R would also inhibit STING-mediated IFN induction since IRF3 activation occurs downstream of both cGAS-STING and RLRs. The same study^24^ also showed that MGF505-2R inhibits the activation of an IFNβ luciferase reporter in cells overexpressing TBK1, thus bypassing STING and supporting our results. This also indicates that MGF505-2R may use multiple mechanisms to impact on IFN induction. Similarly, MGF505-3R was shown to interact with several components of the IFN induction pathway, namely cGAS, TBK1 and IRF3^25^. In this study, MGF505-3R also inhibited an IFNβ luciferase reporter following overexpression of IRF3, corroborating our data.

The CRL complex is a group of cellular E3 ligase machinery that catalyses the ubiquitination of target proteins for 26S proteasomal degradation. Five classes of protein binding motifs are capable of recruiting and acting as a platform for the CRL in a modular fashion; including the F-Box, SOCS-Box and VHL-Box^28,29^. The F-Box motif is commonly observed in some poxviruses proteins and is thought to act in a similar manner to that of cellular counterparts by utilising the CRL machinery to target substrate proteins for degradation^47–50^. Typically, these motifs are found at the C-terminus in host proteins. The closely related ~40 residue VHL-Box and SOCS-Box motifs consist of two distinguishable regions, termed BC-Box and Cul-Box. Unlike F-Box proteins, which recruit SKP1, Cul1, and RBX1 for the formation of the CRL; SOCS proteins recruit the EloB/EloC heterodimer complex via the BC-Box, and the Cul5 scaffold protein driven by the Cul5-Box motif. The VHL-Box motif is distinguished from the SOCS-Box by the recruitment of Cul2 via the Cul2-Box. Here, we identified the presence of a CRL-binding VHL/SOCS-Box like motif at the N-terminus of the MGF505 proteins. Importantly, MGF505-1R was able to co-immunoprecipitate with EloB and Cul5, but not Cul2, suggesting the motif in the MGF505-1R protein is acting as a SOCS-Box. This SOCS-Box like motif was shown to play a crucial role for the ability of MGF505-1R to inhibit the IRF3 and NFκB pathways. We observed a clear reduction in inhibition of both pathways when key residues of the full motif were mutated, whereas the mutation of the BC-Box or Cul-Box alone had much less impact on IRF3-dependent activation. This suggests that the full motif is required for this inhibitory activity. Interestingly, the orthopoxviruses ANK/BC proteins characterised by Odon and colleagues^51^, rely only on the BC-Box to recruit Cul2 and inhibit both IRF3 and NFκB pathways.

The observed inhibition of both IRF3 and NFκB pathways at a level below that of transcription factor activation suggests either dual functionality of the MGF505 proteins in inhibiting both of these pathways and/or a common downstream target. Binding of transcription factors to the IFNβ promoter leads to the formation of the higher order nucleoprotein enhanceosome complex, facilitating the recruitment of TFIIB, and subsequently RNAPII (including largest subunit B1 (Rpb1)) via CBP/p300, ultimately leading to the commencement of transcription^52–54^. CBP/p300 are versatile co-activators. They offer a promiscuous approach to the induction of transcription as they are capable of forming complexes with a wide variety of transcription factors, including NFκB, IRF3, IRF7, AP-1, c-Jun, C-Fos amongst others^43–46,55,56^. Importantly, the interaction between p65 or IRF3 and CBP/p300, is crucial in increasing transcription factor-dependent promoter activation^43,44^. Targeting of p300/CBP has been proposed as a mechanism used by a number of viruses to inhibit host signalling pathways, either by preventing transcription factor interaction or by displacing the cofactor from the promoter^57–61^. ASFV immunomodulatory proteins A238L, and D129L have been shown to act upon these cofactors^57,62^. Granja et al. reported that A238L inhibits transcriptional activation by acting upon the amino terminus of p300 to block interaction with PKC coactivator. This modulates its autoacetylation function, ultimately inhibiting the transcriptional activation of c-Jun, NF-ATc2 and NFκB^58^. D129L inhibited type-I IFN and ISG expression by interfering with the IRF3-p300 interaction post IRF3 transcriptional factor activation, in turn supressing the *IFN-B* promoter^62^. Although we were unable to show a direct interaction between p300 and MGF505-1R, the wild-type ASFV MGF505-1R protein, and to a lesser extent its SOCS-box mutants, clearly reduced the amount of endogenous p300. However, it is curious that despite the extensive homology between p300 and CBP, the viral protein appears to be specifically targeting p300. Despite the shared domain architecture and sequence similarity of roughly 70%, the proteins share much lower homology outside the functional domain regions^63^. MGF505-1R may target p300 via these regions but this remains to be elucidated. As the pig and human p300 share 95% amino acid identity, it is expected that MGF505-1R would have a similar impact on porcine-derived cells.

The observations described in this manuscript should be validated in ASFV target cells. However, ASFV predominantly infects primary porcine macrophages, which are not amenable to transfection. In addition, the absence of an antibody that specifically recognises individual MGF505 proteins such as MGF505‑1R currently prevents us from assessing these findings in ASFV‑infected cells. Our previous results^10^ revealed that MGF360 and 505 proteins do not act synergistically to inhibit the induction of type I IFN. Indeed, the deletion of MGF505-1R alongside MGF360-12L attenuated ASFV genotype II Georgia2007/1 in pigs and increased secretion of IFN-α by PBM cells, but at lower levels than the MGF360-12L single deletion. Interestingly, and despite the results presented here in transfected cells, the deletion of MGF505-1R alone did not have an impact upon IFN-α secretion in infected cells^10^. This again highlights the complexity of the mechanisms deployed by ASFV to manipulate IFN induction, with high levels of redundancy regarding host factors targeted by the virus proteins (reviewed by Netherton et al.^64^), perhaps compensating for the absence of some genes but not others. In summary, we have demonstrated the presence of a functional CRL recruitment motif within an ASFV protein, and the potential of MGF505-1R to use this machinery for the targeted degradation of the transcription co-factor p300. This provides evidence that, like other viruses, ASFV takes advantage of the host CRL machinery to control key host antiviral pathways. One exciting possibility is that other members of the MGF505 may also recruit the CRL complex to target different host proteins for degradation. Indeed, others have recently found that not only MGF505 proteins but also MGF360 proteins recruit the CRL system to leverage the host ubiquitin-proteasome pathway. Interestingly, while MGF505 proteins recruit Cul5, as we have shown here, MGF360 can recruit either Cul2 or Cul5 to target a variety of host proteins (Glassman et al., submitted). Collectively, this indicates that ASFV MGF505 (and MGF360) proteins use an evolutionary conserved mechanism to degrade proteins, whilst retaining their specificity by impacting on different host targets and pathways, possibly through their putative ankyrin repeats domains.

## Methods

### Cell culture

HEK293T, HeLa, and A549 cells were supplied by The Pirbright Institute’s internal cell bank (obtained from the European Collection of Authenticated Cell Cultures - ECACC) and maintained in DMEM Glutamax (Gibco) supplemented with 10% foetal calf serum (Life Science Production), and 100 U/mL penicillin streptomycin (Gibco).

### Plasmids and transfections

Gal4-luciferase reporters (pGal4.Luc) and stimulus constructs (pGal4-p65, pGal4.IRF3, pGal4-147) were generously supplied by Steve Goodbourn (City St George’s, University of London). pJAC.LacZ (βGal) was a kind gift from Michael Baron (The Pirbright Institute, UK). Constructs containing host genes in various vectors for expression, pMC160.HA, and vectors required for luciferase assay systems (firefly luciferase under the control of NFκB and IRF3 regulatory elements (pNFκB.Luc, pIRF3.Luc) and renilla luciferase (pTK.Renilla)), were a kind gift from Joanna Shisler (University of Illinois, USA). pCMV5.p300.HA was a generous gift from Tony Kouzarides (University of Cambridge). pNpro.GFP was kindly supplied by Julian Seago (The Pirbright Institute, UK). pcDNA3.1 vector containing Cul2.Myc, Cul5.Myc, T7.EloC, and HA.EloB were purchased from Addgene Plasmid Repository (Addgene). Constructs expressing MGF505-1R, 2R, and 3R for use in mammalian expression systems were designed based on the sequence from the Georgia07/1 isolate (GenBank: FR682468.2) and synthesised by GeneArt® into pcDNA3.1 vector to include a fusion to a C-terminus V5 (GKPIPNPLLGLDST) tag. Plasmid constructs containing mutant variants of ASFV genes (MGF505-1R.mSOCS.V5, MGF505-1R.mBC.V5, MGF505-1R.mCUL.V5) in pcDNA3.1 vector were synthesised by GenScript. All the constructs containing ASFV genes were codon optimised for expression in human cells. Cells were transfected with plasmids using TransIT-LT1 (Mirus Bio) and incubated for 24 hours before stimulation or lysis.

### Luciferase reporter assays

HEK293T cells were seeded at a density of 1.25 × 10^5^ cells per well (24 well plate) and were transfected with firefly luciferase reporter plasmid pNFκB.Luc (100 ng/well) or pIRF3.Luc (100 ng/well), and endogenous control plasmids pTK.Renilla (10 ng/well) or pLacZ (25 ng/well). Cells were co-transfected with either the empty vector (pcDNA3.1) or vector expressing viral proteins (350 ng/well).

In assays stimulated by exogenous protein expression, cells were transfected with plasmids expressing the following proteins: IKKα.flag, IKKβ.flag, flag.TBK1, IRF3-5D, or Gal4-IRF3-65-427 (100 ng/well), or p65-GFP or Gal4DBD-p65 (5 ng/well) and incubated for 24 h.

To ensure consistency across all conditions, total DNA per well was equalised with empty pcDNA3.1 vector. Otherwise, cells were stimulated with TNFα (20 ng/mL), IL-1β (10 ng/mL) for 6 h, or Sendai virus Cantell strain (200 HAU/well, Charles River) for 24 h. Each technical experiments, performed at least three times, contained biological duplicates of unstimulated and stimulated conditions.

Cells were lysed in 100 μL 1X Passive Lysis Buffer (Promega) for dual-luciferase assays, or 100 μL 1X Reporter Lysis Buffer (Promega) for β-Galactosidase assays, for 15 min with shaking followed by freeze thawing at −20 °C. Dual-luciferase reagents (Luciferase Buffer II and Stop-Glo buffer) were used to measure luciferase activity (Promega), or Luciferase assay Buffer I (Promega) and GalactonPlus (Applied Biosystems) for β-Galactosidase assays. Plates were read via a Cytation 3 dispenser plate reader (BioTek).

### Western blotting and co-immunoprecipitation

For western blotting, transfected HEK293T cells were lysed on ice, in RIPA buffer (ThermoFisher) containing 1× Halt Protease and Phosphatase Cocktail inhibitor with EDTA (ThermoFisher) and incubated for 10 min. Samples were diluted to 1× in Pierce™ Lane Marker Reducing Sample Buffer (ThermoFisher) and incubated for 30 min at room temperature before SDS-PAGE.

For co-immunoprecipitation assays, transfected HEK293T cells were incubated in 2 mL of cross-linking agent dithiobis succinimidyl propionate (DSP) (Thermofisher) dissolved in anhydrous DMSO (Sigma Aldrich) to a final concentration of 2 mM in PBS on ice for 2 h, and 1 M Tris HCl at pH 7.5 (Invitrogen) was added at a final concentration of 20 mM for 15 min to quench the reaction. Cells were then incubated in 200 μL 1× IP buffer supplemented with 1× Halt Protease and Phosphatase Cocktail inhibitor without EDTA (extraction buffer) for 15 min before being scraped off. Samples were spun down at 20,000 × *g* at 4 °C for 10 min. Ten percent of the sample supernatant was retained as whole cell lysate input, and the remaining lysate was used for CoIP using the ThermoFisher Epoxy270 Dynabead CoIP Kit protocol to manufacturer’s instructions. All steps were performed on ice. Samples were incubated with rotation with 1.5 mg magnetic Epoxy270 Dynabeads™, previously covalently coupled to antibody, at 4 °C for 45 min. Prior to SDS-PAGE, samples were diluted to 1× in Pierce™ Lane Marker Reducing Sample Buffer (ThermoFisher) containing DTT and incubated at 100 °C for 5 min.

Proteins were transferred to 0.2 or 0.4 µm PVDF membranes using Mini Trans-Blot® Electrophoretic Transfer Cell (BioRad) in ice cold 1× TGS-SDS with 20% methanol at 100 V for 90 min. Membranes were blocked in 5% milk TBST for 1 h at RT, incubated with primary antibody diluted in 5% BSA TBST or 5% milk TBST overnight at 4 °C. Antibodies against the following proteins were used: Cullin-2, 51-1800 (1:250, Invitrogen), Cullin-5, EPR14725 (1:1000, AbCam), Elongin B, EPR10441 (1:1000, AbCam), Elongin C, ab226831 (1:1000, AbCam), HA-tag (1:1000, Roche), IκBα, 9242 (1:1000, Cell Signalling), IKKβ, D30C6 (1:1000, Cell Signalling), IRF3, D83B9 (1:1000, Cell Signalling), Myc-Tag, 9B11 (1:1000, Cell Signalling), p300, D8Z4E (1:1000, Cell Signalling), phospho-p65 (S536), 93H1(1:1000, Cell Signalling), TBK1, D1B4 (1:1000, Cell Signalling), V5-Tag (1:1000, BioRad), V5-Tag HRP (1:2000, Bethly Inc.), α-Tubulin, DM1A (1:1000, Cell Signalling) and β3-Tubulin (1:1000, Invitrogen). Final incubation with species appropriate secondary antibodies, goat anti-mouse IgG (1:1000, Invitrogen), mouse anti-rabbit IgG (1:2000, Cell Signalling) and goat anti-rat (1:500, DAKO), in 5% Milk TBST was performed for 1–2 h at RT. Pierce™ ECL Plus Western Blotting Substrate (ThermoFisher) was used for imaging.

### Immunofluorescence

HeLa or A549 cells were seeded on glass cover slips at a density of 5 × 10^4^ cells per well (24 well plate). Cells were transfected with 450 ng per well of relevant plasmids as described above. When described in the text, HeLa cells were stimulated with TNFα (20 ng/mL) for 20 min, whereas A549 cells were stimulated with SeV (200 HAU/mL) for 4 h, before fixation in 4% PFA and permeabilization in 0.2% Triton X-100. Cells were blocked in 2% BSA for 1 h at RT. Cells were incubated with the following primary antibodies: anti-IRF3, 1HCLC (1:100, Invitrogen), anti-p65, D14E12 (1:400, Cell Signalling), p300, D8Z4E (1:500, Cell Signalling) and anti-V5 tag (1:350, BioRad) in 0.2% BSA for 1 h. Cells where then incubated with secondary antibodies diluted in the same buffer: goat anti-mouse Alexa Fluor 568 and goat anti-rabbit Alexa Fluor 488 (both at 1:500, Life Technologies). DAPI was used for DNA staining. Cells were visualised using a Leica TCS SP8 confocal microscope (Leica) and processed with LASX or LAS AF Lite confocal software (Leica). For quantification of the p300 fluorescence signal, isosurface renderings were generated in Imaris (version 10.1, Oxford Instruments) and classified as either V5‑positive or V5‑negative. The mean p300 fluorescence intensity for each isosurface was then exported and subsequently analysed in GraphPad Prism.

### Structural modelling of protein complexes

Protein complexes were generated using AlphaFold3^39^, with the following *sus scrofa* protein sequences: cullin-5 (XP_005667355.1), EloB (XP_003124794.2) and EloC (XP_020944849.1). Visualisation and structure analyses were performed using UCSF ChimeraX 1.9^65^.

### Statistical analysis

GraphPad Prism Version 8.4.2 (GraphPad Software) was used to for statistical analysis. Two-way ANOVA Dunnett’s or Tukey’s multiple comparison tests were applied to data with two independent variables, such as unstimulated or stimulated conditions; ****p ≤ 0.0001, ***p ≤ 0.001, **p ≤ 0.01, *p ≤ 0.05.

## Supplementary information

Supplementary Information

## Data Availability

The datasets generated and/or analysed during the current study are available in “figshare” repository, at https://figshare.com/s/d695d854529d9d3102f5.
