# Supplementary material for: The African swine fever virus MGF505-1R protein recruits the cullin-RING-ligase machinery to promote p300 degradation
Source: Npj Viruses. 2026 May 2;4:32. doi: 10.1038/s44298-026-00191-8 (PMC13342108; doi:10.1038/s44298-026-00191-8)
Supplement: Supplementary file 1 — Supplementary Information [file 44298_2026_191_MOESM1_ESM.pdf]

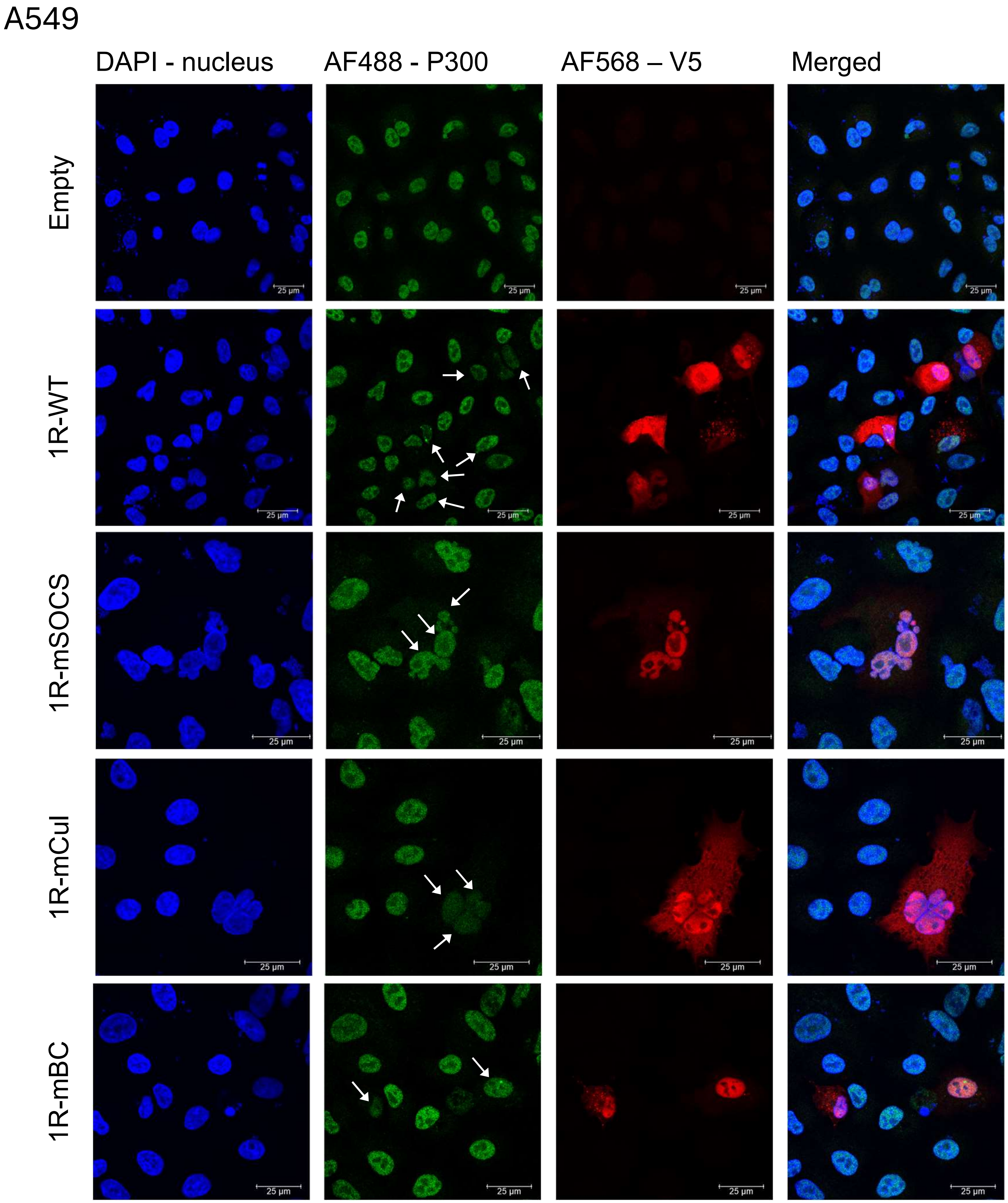
A549
DAPI - nucleus
AF488 - P300
AF568 – V5
Merged
Empty
1R-WT
1R-mSOCS
1R-mCul
1R-mBC
25 μm

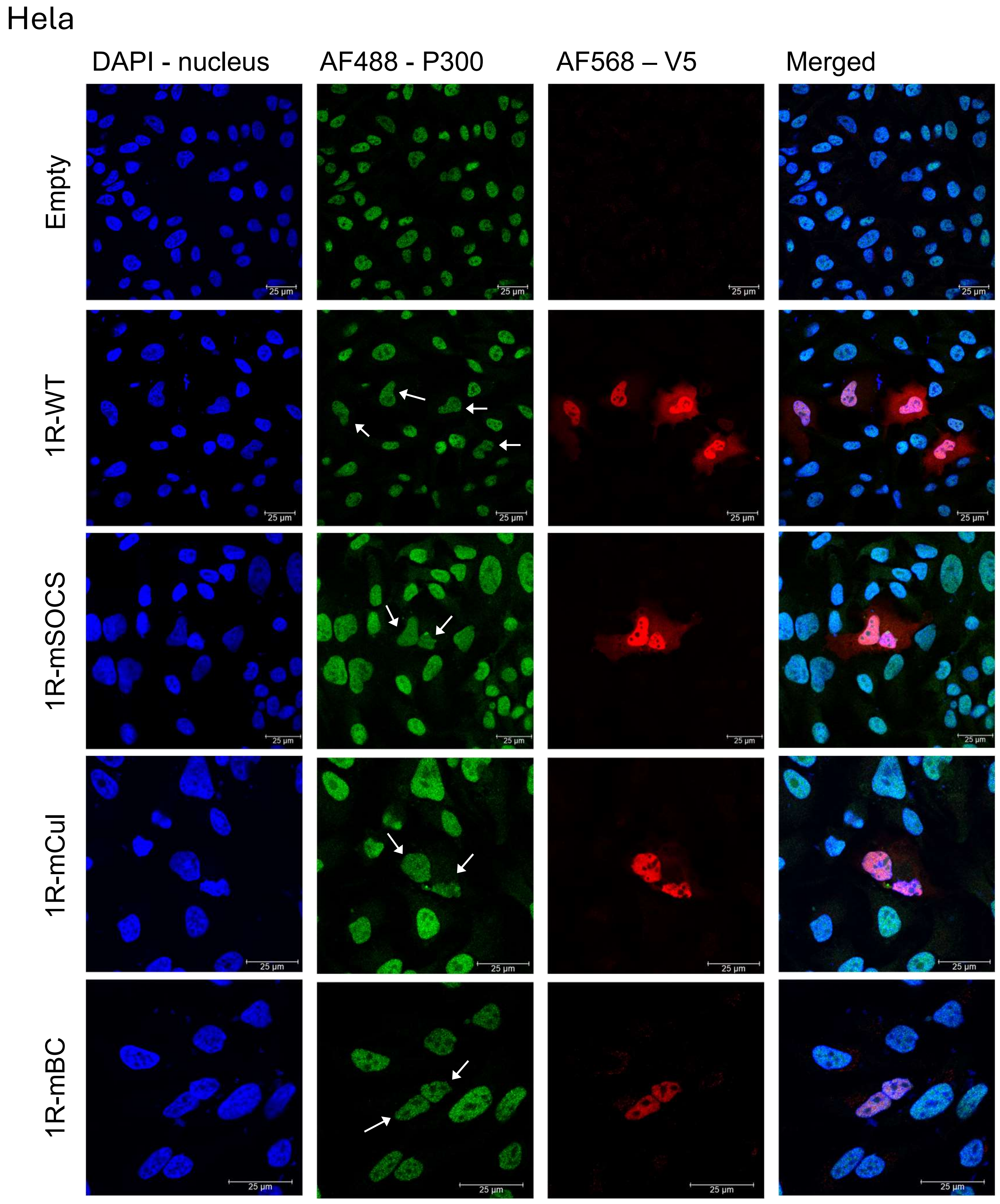
Hela
DAPI - nucleus
AF488 - P300
AF568 – V5
Merged
Empty
1R-WT
1R-mSOCS
1R-mCul
1R-mBC
25 µm

HEK293T

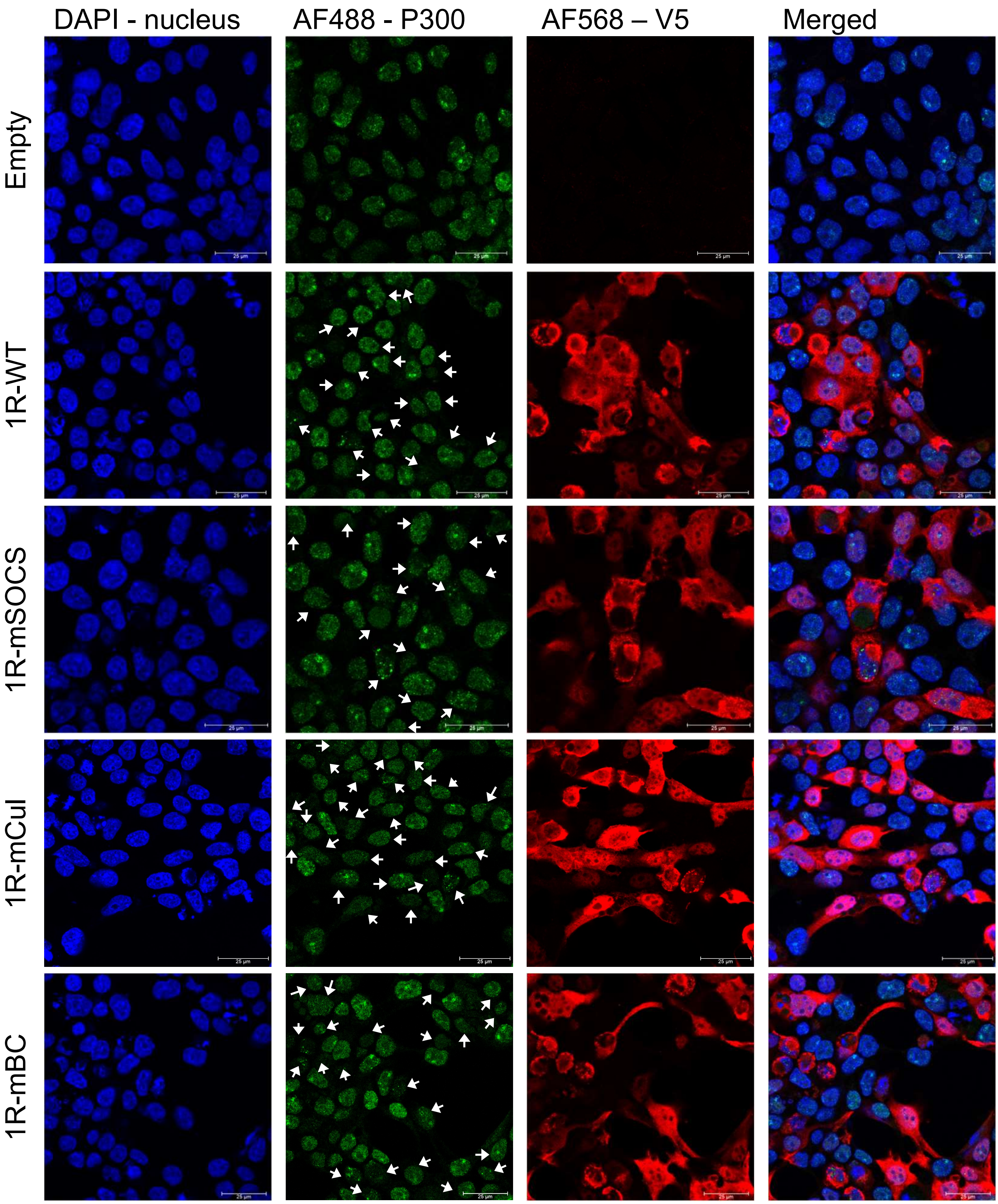

Supplementary Figure 1. Expression of p300 on cells transfected with MGF505-1R or its mutant variants. Confocal immunofluorescence showing A549, Hela or HEK293T cells transfected with plasmids expressing WT MGF505-1R, or its mutant variants MGF505-1R.mSOCS.V5, MGF505-1R.mBC.V5 and MGF505-1R.mCUL.V5, and probed for V5 (red), and endogenous p300 (green). Blue shows DAPI nuclear staining. White arrows indicate V5 positive cells.
